# gcType: a high-quality type strain genome database for microbial phylogenetic and functional research

**DOI:** 10.1093/nar/gkaa957

**Published:** 2020-10-29

**Authors:** Wenyu Shi, Qinglan Sun, Guomei Fan, Sugawara Hideaki, Ohkuma Moriya, Takashi Itoh, Yuguang Zhou, Man Cai, Song-Gun Kim, Jung-Sook Lee, Ivo Sedlacek, David R Arahal, Teresa Lucena, Hiroko Kawasaki, Lyudmila Evtushenko, Bevan S Weir, Sarah Alexander, Dlauchy Dénes, Somboon Tanasupawat, Lily Eurwilaichitr, Supawadee Ingsriswang, Bruno Gomez-Gil, Manzour H Hazbón, Marco A Riojas, Chatrudee Suwannachart, Su Yao, Peter Vandamme, Fang Peng, Zenghui Chen, Dongmei Liu, Xiuqiang Sun, Xinjiao Zhang, Yuanchun Zhou, Zhen Meng, Linhuan Wu, Juncai Ma

**Affiliations:** Microbial Resource and Big Data Center, Institute of Microbiology, Chinese Academy of Sciences, Beijing 100101, China; World Data Center for Microorganisms, Beijing 100101, China; Microbial Resource and Big Data Center, Institute of Microbiology, Chinese Academy of Sciences, Beijing 100101, China; World Data Center for Microorganisms, Beijing 100101, China; China-Thailand Joint Laboratory on Microbial Biotechnology, Beijing 100190, China; Microbial Resource and Big Data Center, Institute of Microbiology, Chinese Academy of Sciences, Beijing 100101, China; World Data Center for Microorganisms, Beijing 100101, China; National Institute of Genetics, Yata, Mishima 411-8540, Japan; Japan Collection of Microorganisms (JCM)/ Microbe Divion, RIKEN BioResource Center, Koyadai 3-1-1, Tsukuba, Ibaraki 305-0074, Japan; Japan Collection of Microorganisms (JCM)/ Microbe Divion, RIKEN BioResource Center, Koyadai 3-1-1, Tsukuba, Ibaraki 305-0074, Japan; China General Microbiological Culture Collection Center (CGMCC), Institute of Microbiology, Chinese Academy of Sciences, Beijing 100101, China; China General Microbiological Culture Collection Center (CGMCC), Institute of Microbiology, Chinese Academy of Sciences, Beijing 100101, China; Korean Collection for Type Cultures (KCTC), Korea Research Institute of Bioscience and Biotechnology (KRIBB), 181 Ipsin-gil, Jeongeup-si, Jeollabuk-do, 56212, Republic of Korea; Korean Collection for Type Cultures (KCTC), Korea Research Institute of Bioscience and Biotechnology (KRIBB), 181 Ipsin-gil, Jeongeup-si, Jeollabuk-do, 56212, Republic of Korea; Czech Collection of Microorganisms, Masaryk University, Kamenice 5, building A25, 625 00 Brno, Czech Republic; Colección Española de Cultivos Tipo (CECT), and Departamento de Microbiología y Ecología, University of Valencia, 46100 Burjassot (Valencia), Spain; Colección Española de Cultivos Tipo (CECT), and Departamento de Microbiología y Ecología, University of Valencia, 46100 Burjassot (Valencia), Spain; NITE Biological Resource Center (NBRC), National Institute of Technology and Evaluation, 2-5-8 Kazusakamatari, Kisarazu, Chiba 292-0818, Japan; All-Russian Collection of Microorganisms (VKM), G.K. Skryabin Institute of Biochemistry and Physiology of Microorganisms RAS, Pushchino, Moscow region 142290, Russia; Mycology & Bacteriology Systematics, Manaaki Whenua – Landcare Research, Auckland, New Zealand; National Collection of Type Cultures (NCTC), Public Health England (PHE), UK; National Collection of Agricultural and Industrial Microorganisms, Faculty of Food Science, Szent István University, H-1118, Budapest, Somlói út 14-16, Hungary; Faculty of Pharmaceutical Sciences, Chulalongkorn University (PCU), Bangkok 10330, Thailand; China-Thailand Joint Laboratory on Microbial Biotechnology, Beijing 100190, China; Thailand Bioresource Research Center (TBRC), National Center for Genetic Engineering and Biotechnology (BIOTEC), National Science and Technology Development Agency (NSTDA), Thailand; China-Thailand Joint Laboratory on Microbial Biotechnology, Beijing 100190, China; Thailand Bioresource Research Center (TBRC), National Center for Genetic Engineering and Biotechnology (BIOTEC), National Science and Technology Development Agency (NSTDA), Thailand; CIAD, A.C., Collection of Aquatic Important Microorganisms (CAIM). AP 711 Mazatlán, Sinaloa, Mexico; American Type Culture Collection(ATCC), 10801 University Boulevard, Manassas, VA 20110, USA; American Type Culture Collection(ATCC), 10801 University Boulevard, Manassas, VA 20110, USA; Biodiversity Research Centre, Thailand Institute of Scientific and Technological Research (TISTR), 35 M 3 Technopolis Khlong 5 Khlong Luang Pathum Thani 12120, Thailand; China Center of Industrial Culture Collection (CICC), Beijing, China; BCCM/LMG Bacteria Collection, Laboratory of Microbiology, Faculty of Sciences, Ghent University, K. L. Ledeganckstraat 35, 9000 Ghent, Belgium; China Center for Type Culture Collection (CCTCC), College of Life Sciences, Wuhan University, Wuhan 430072, China; Microbial Resource and Big Data Center, Institute of Microbiology, Chinese Academy of Sciences, Beijing 100101, China; World Data Center for Microorganisms, Beijing 100101, China; Microbial Resource and Big Data Center, Institute of Microbiology, Chinese Academy of Sciences, Beijing 100101, China; World Data Center for Microorganisms, Beijing 100101, China; Microbial Resource and Big Data Center, Institute of Microbiology, Chinese Academy of Sciences, Beijing 100101, China; World Data Center for Microorganisms, Beijing 100101, China; Microbial Resource and Big Data Center, Institute of Microbiology, Chinese Academy of Sciences, Beijing 100101, China; World Data Center for Microorganisms, Beijing 100101, China; Computer Network Information Center, Chinese Academy of Sciences, Beijing 100190, China; Computer Network Information Center, Chinese Academy of Sciences, Beijing 100190, China; Microbial Resource and Big Data Center, Institute of Microbiology, Chinese Academy of Sciences, Beijing 100101, China; World Data Center for Microorganisms, Beijing 100101, China; State Key Laboratory of Microbial Resources, Institute of Microbiology, Chinese Academy of Sciences, Beijing 100101, China; Microbial Resource and Big Data Center, Institute of Microbiology, Chinese Academy of Sciences, Beijing 100101, China; World Data Center for Microorganisms, Beijing 100101, China; China-Thailand Joint Laboratory on Microbial Biotechnology, Beijing 100190, China; State Key Laboratory of Microbial Resources, Institute of Microbiology, Chinese Academy of Sciences, Beijing 100101, China

## Abstract

Taxonomic and functional research of microorganisms has increasingly relied upon genome-based data and methods. As the depository of the Global Catalogue of Microorganisms (GCM) 10K prokaryotic type strain sequencing project, Global Catalogue of Type Strain (gcType) has published 1049 type strain genomes sequenced by the GCM 10K project which are preserved in global culture collections with a valid published status. Additionally, the information provided through gcType includes >12 000 publicly available type strain genome sequences from GenBank incorporated using quality control criteria and standard data annotation pipelines to form a high-quality reference database. This database integrates type strain sequences with their phenotypic information to facilitate phenotypic and genotypic analyses. Multiple formats of cross-genome searches and interactive interfaces have allowed extensive exploration of the database's resources. In this study, we describe web-based data analysis pipelines for genomic analyses and genome-based taxonomy, which could serve as a one-stop platform for the identification of prokaryotic species. The number of type strain genomes that are published will continue to increase as the GCM 10K project increases its collaboration with culture collections worldwide. Data of this project is shared with the International Nucleotide Sequence Database Collaboration. Access to gcType is free at http://gctype.wdcm.org/.

## INTRODUCTION

Microorganisms are considered the most abundant organisms in the world. It is estimated that ∼4–6 × 10^30^ prokaryotic cells exist on Earth, comprising a biomass of 350–550 × 10^15^ g of carbon (1). The total number of prokaryotic species is up to 10^9^ (2). Approximately 1800 bacterial and archaeal species names were published in approved lists of bacterial names in 1980 (3). Thereafter, names published in original articles or in the ‘Validation Lists’ of the *International Journal of Systematic and Evolutionary Microbiology* (IJSEM) have been validated. As of September 2020, this number increased to 16 763.

The description of a prokaryotic species needs to designate its type strain, whose phenotypes and genotypes are often well characterised and described. 16S rRNA gene and whole-genome sequences derived from type strains, together with phenotypic and chemotaxonomic characteristics are used for taxonomic identification. Thus, type strains are critical references for the characterisation of species and the identification of isolates and strains for taxonomic purposes (4). Currently, the type strains from the 16 763 listed species are available as 67 331 catalogue numbers from over 130 culture collections.

For several decades, it has been recognised that the complete deoxyribonucleic acid (DNA) sequence of a species would be the standard reference to determine their phylogeny, which in turn determines their taxonomic classification (5). With the increasing availability of genome sequences, genome-based methods such as average amino acid identity, average nucleotide identity (ANI) and digital DNA–DNA hybridization (dDDH) have been developed as important measurements for prokaryotic taxonomy (6–8). Since January 2018, IJSEM requires authors of new taxa to provide genome sequences with descriptions of the novel taxa for their manuscripts to be eligible for publication. Data on 16S rRNA similarity, overall genome similarity or distance and phenotypic and physiological information are used in combination to identify a new species (9).

Another essential element of microbial taxonomy is the correct assessment of phylogenetic relationships via the reconstruction of phylogenetic trees. Although phenotypic, chemotaxonomic and genotypic information are useful for identifying microorganisms, such information is often insufficient for reconstructing accurate phylogenies. The increasing availability of microbial genome sequences allows for more comprehensive and accurate depictions of phylogenetic relationships to study the origin and evolution of prokaryotic organisms.

Because of their genomic and hence metabolic and functional diversity, microorganisms serve as ideal models for biotechnology studies. Combined with comprehensive phenotypic and physiological information, genome sequences of type strains enable the connection of genes with functions and provide insights into the metabolic and functional potential of microorganisms. Therefore, accruing data on genomes of microorganisms will greatly promote biotechnology studies.

Given the immense efforts of microbiologists and community sequencing projects such as The Genomic Encyclopedia of Bacteria and Archaea (GEBA) (10), the number of publicly available genome sequences of type strains continues to increase rapidly. Currently, there are >12 000 genome sequences in the International Nucleotide Sequence Database Collaboration (INSDC). However, a large number of microbial type strains remain to be sequenced. Therefore, the World Data Centre for Microorganisms (WDCM) GCM 10K project (11) is cooperating with culture collections across the world to fill the current gap in whole-genome databases for validly published species as well as with IJSEM to provide free sequencing and genome annotation services for newly described species (12).

With increasing microbial genomic information, the databases and servers which host and analyse these data continue to expand. The Genomes Online Database (GOLD) (13) in conjunction with the Integrated Microbial Genomes (IMG) (14) provide a comprehensive catalogue of microbial genomes and platform for the analysis of microbial genomes and microbiomes. The Type (Strain) Genome Server (15), which is connected to the comprehensive prokaryotic metadata resources BacDive (16) and LPSN (17), is considered a high-throughput web server for genome-based prokaryotic taxonomy. However, there is still a need for a database that provides not only up-to-date type strains and the associated comprehensive genome information but also user-friendly searchable and comparable functions.

To facilitate access to and maximise the value of genome sequences of type strains, the GCM 10K type strain sequencing project developed the gcType platform. This platform integrates publicly available information from other databases along with the sequencing efforts of GCM 10K project according to strict quality control standards, followed by a powerful standard data-processing pipeline to yield a high-quality reference database that provides web-based data analysis pipelines for genomic analysis and genome-based species identification. Moreover, it associates taxonomic, phenotypic and physiological information with the type strains to enable users to conduct comprehensive genomic and functional analyses. On the whole, gcType is a unique and useful resource that permits microbial taxonomists and microbiologists to gather up-to-date information on microbial type strain sequences.

## DATABASE DESIGN AND IMPLEMENTATION

### gcType portal and search functions

**Figure 1.**
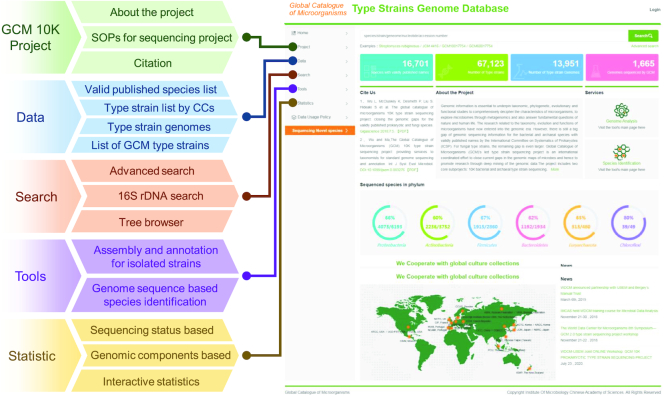
Features of the gcType portal: a map of the main pages and list of the subordinate pages on the gcType website.

Users of the platform can query the database and perform genomic analyses using the gcType portal. gcType is the GCM 10K sequencing project data portal for the dissemination of information and publication of updated sequencing results. It currently provides multiple, flexible search functions for users to explore its resources (Figure 1). A text-based advanced search option allows users to conduct cross-genome searches through a single or combination of metadata information. The input query retrieves all data containing the corresponding keywords in the metadata fields, such as the sequencing status, library or contig or scaffold numbers.

All validly published species have been mapped onto the National Center for Biotechnology Information (NCBI) taxonomy ID (18), and all genome sequences have been mapped onto the Genome Taxonomy Database (GTDB) system using GTDB-Tk (19). Users can browse through these taxonomic trees to search for sequenced and un-sequenced species. Sequence-based searches against pre-generated type strain 16S rRNA sequence databases using the Basic Local Alignment Search Tool (BLAST) are provided as well. The resultant hits of the query sequences are displayed as alignments and links to the matched type strains.

A statistics page displaying tables with genomic characteristics provides gcType users with an overview of the diversity of microbial genomic data. In particular, these tables feature the guanine-cytosine (GC) content, average genome size, number of predicted genes and functional annotation results among different phyla. Interactive interfaces allow users to further explore the features of various taxonomic groups.

### Integrated information for type strains

**Figure 2.**
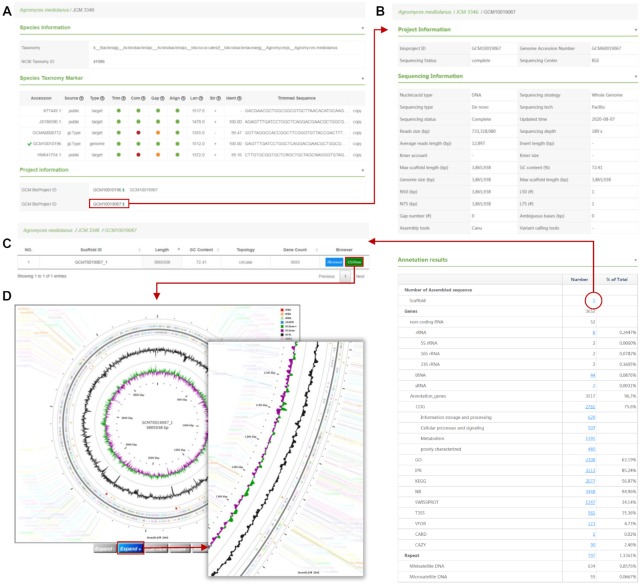
Comprehensive information about a type strain and its associated genome sequences. (**A**) Species information and marker genes. The NCBI taxonomy ID is linked to the NCBI taxonomy database. (**B**) Project and sequencing information. (**C**) Genome annotation results with the associated statistics. (**D**) Interactive circular view of type strain genome.

Comprehensive taxonomic, phenotypic, physiological and genomic information is organised by type strain species (Figure 2A-2D). The taxonomic status, 16S rRNA gene sequence and NCBI taxonomy ID are provided. One or several ‘genome sequence project’ pages are linked with the type strain page. For genome sequences extracted from public resources, GenBank (20) or GOLD ‘Bioproject’, ‘Biosample’ and ‘Assembly IDs’ are provided as links to the original sites in GenBank. Simultaneously, a ‘GCM project number’ is assigned to the sequence and linked to the annotation results generated by the GCM microbial genomic annotation pipeline. Metadata from the genome sequences (e.g. library, N50 and GC content) as well as annotation results of the genome are listed by hits from various reference databases such as COG (21), KEGG (22) and CARD (23). Finally, It also incorporates open-source web applications: JBrowse (24), a linear genome viewer, and CGView (25), a circular genome viewer.

### Data sources

#### Type strain information

Lists of validly published species, a list of type strains, a list of type strains by culture collection, and a comprehensive list of 16S rRNA gene and genome sequences are provided. New prokaryotic taxa were considered validly published only if their names were published in the *International Journal of Systematic Bacteriology* (until October 1999) or the renamed IJSEM (from January 2000 to present). Names published in an original article, or the ‘Validation Lists’ are considered to be valid (26). The list of species with validly published names were manually collected from IJSEM novel species articles and the validation lists. All data were collected until September 2020. IJSEM and the International Committee on Systematics of Prokaryotes require the type strains of new taxa to be deposited in at least two recognised culture collections in two countries. The deposition of type strains and related metadata information has been described in IJSEM articles. For candidate type strains of novel species, taxonomists who request free genome sequencing services from WDCM are asked to provide detailed metadata of the type strains before WDCM formally accepts their proposal. The metadata fields are following the description recommended by the minimum information about a genome sequence (MIGS) specification (27).

#### 16S rRNA gene sequence data

**Figure 3.**
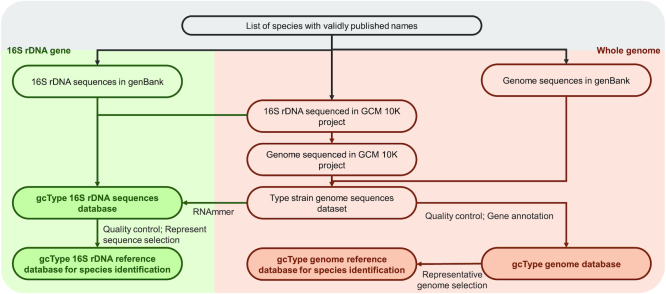
Schematic representation of gcType for data processing: Two sources of 16S rRNA gene sequences are integrated to form the gcType 16S rRNA gene database. Sequence data is further aligned and trimmed to form a reference database for a new species identification pipeline. Publicly available genome sequences and GCM 10K genome sequences are processed by the GCM microbial genome annotation pipeline to form a type strain genome database. The assembled genomes from these two sources are integrated after quality control to form a type strain genome reference database for a new species identification pipeline.

16S rRNA gene sequences similarity comparison remains the initial step in the identification of prokaryotic species workflow; therefore, a high-quality 16S rRNA gene sequence database of species with validly published names is fundamental for conducting taxonomy studies. Some reference databases for 16S rRNA gene sequences, e.g. EzBiocloud (28), SILVA (29) and RDP (30), provide online resources based on different data integration strategies and filtering criteria (31). Because gcType is designed for type strain-based taxonomic studies, we collected 16S rRNA gene sequences from two sources. The first contains 16S rRNA gene sequences from publicly available resources, including 16S rRNA genes or sequences derived from completed or partial genomes by RNAmmer (32). Their accession numbers were obtained from GenBank, and the associated sequence data were then extracted. Second, for the GCM 10K sequencing project, prior to proceeding with whole-genome sequencing, submitted strains are validated via 16S rRNA sequencing. The sequences obtained from the GCM 10K project were added to the database to allow the quality of type strains to be examined because a small percentage the strains are incorrect, likely due to deposition or preservation errors. Data were further filtered and evaluated based on quality control criteria such as sequence length, number of ambiguous bases, completeness and the accuracy of the metadata. After alignment with sequences in infernal (33) and Rfam (34) databases and trimming of the 5′ and 3′ ends the two sources of 16S rRNA gene sequences were integrated to construct a reference dataset for the new species identification pipeline. Evaluation scores related to data quality are displayed on the webpage and can be selected by users. Data processing is described in Figure 3.

#### Genome sequencing information and sequence data

gcType publishes genome sequences and their annotation results from the GCM 10K sequencing project, which utilises the next-generation sequencing (NGS) platform. To improve the quality of these sequences, we employ third-generation sequencing (TGS) technologies (we are currently using Pacific Biosciences) as a complementary, allowing for the generation of completed or nearly completed bacterial genomes. Importantly, this combination strategy using second- and third-generation sequencing platforms is used for genome sequences that have been poorly assembled in the second-generation sequencing platform alone (>50 contigs).

Raw sequence data that pass strict quality control criteria are run through the GCM microbial genome annotation pipeline. Annotated sequences are then added to the gcType type strain genome database.

Besides the genome sequences in the GCM 10K project, publicly available genome sequence data are extracted from GenBank via their unique type strain numbers. Genome sequences with gene prediction results provided by GenBank in FASTA format were used to perform a GCM microbial genome annotation pipeline; genome sequences without gene prediction results were used to perform a genomic component analysis followed by annotation using the GCM microbial genome annotation pipeline. These newly annotated type strain sequences are then incorporated into the gcType Type strain genome database.

To create a non-redundant set of representative genomes, genome sequences are further filtered by the number of contigs, genome sizes and N50 statistics. Sequences with number of contigs larger than 500 are filtered in the reference database. A single, high-quality representative sequence from each species is selected to create the reference dataset for the new species identification pipeline. Scores pertaining to the quality of the genome are displayed on the webpage and can be selected if there is more than one sequence. A schematic representation of data processing is shown in Figure 3.

As of September 2020, gcType contains 13 962 prokaryotic type strain genomes, from which 1049 were produced by the GCM 10K sequencing project.

### Database design

gcType uses the open-source MySQL relational database management system to manage the data. This database contains tables that are continuously updated with information on species’ taxonomy and nomenclature. Phenotypic and physiological metadata descriptions are linked with proper taxa status. The sequencing projects and their associated metadata and annotation results are linked to the type strains. Scaffolds and predicted features from genomic analyses are related to the assembled genome.

gcType follows an identifier system similar to that of INSDC, in which a ‘GCM Biosample’ number is assigned to each type strain and a ‘GCM Bioproject’ number to each sequencing project. For type strains that have been sequenced multiple times and thus have several sets of sequencing results, different ‘GCM Bioproject’ numbers are assigned to each sequencing effort, a ‘locus tag’ is assigned to each predicted gene and an ‘assembly number’ is assigned to the assembled genome.

## DATA ANALYSIS PIPELINES

Currently, owing to difficulties in using multiple bioinformatics tools and programming scripts for in-house analyses, taxonomists often rely on commercial sequencing and data analysis services to generate genome sequences. However, due to a wide range of data models, annotation pipelines and versions of reference databases, the results of analysing the same genome vary greatly. Functional assignments generated from the same gene using different resources may generate very different results (35).

High-quality valid databases that include the 16S rRNA gene and genome sequences are prerequisites for taxonomic classification and identification. Because these pipelines are designed for taxonomic purposes, only data of type strains with validly published names are included. Therefore, gcType features two reference database integrated pipelines: genome assembly and annotation pipeline and the new species identification pipeline.

### Genome assembly and annotation pipeline

**Figure 4.**
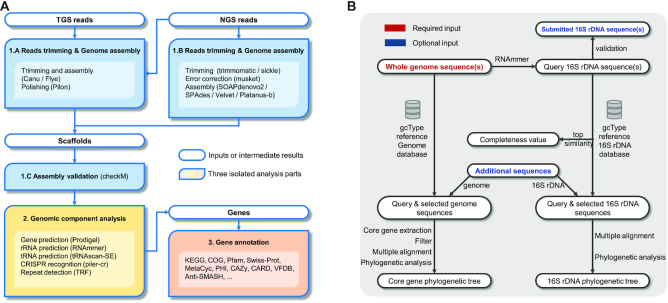
Workflow of gcType data analysis pipelines. (**A**) GCM microbial genome assembly and annotation pipeline. (**B**) New species identification pipeline.

The genome assembly and annotation pipeline comprises three analytical procedures (Figure 4A): (i) processing of raw reads and assembly, (ii) genomic component analysis and (iii) gene annotation.

- **Processing of raw reads and assembly**
- If TGS long reads (PacBio or Nanopore reads) are provided as the input, the raw sequencing reads are trimmed and assembled into contigs or scaffolds using Canu (36) or Flye (37). If NGS short reads (Illumina paired-end reads) are also provided, they will be used to enhance contigs or scaffolds using Pilon (38).
- If only NGS short reads are provided, raw reads are trimmed into clean reads using Sickle (https://github.com/najoshi/sickle) or Trimmomatic (39), corrected using Musket (40) and independently assembled into contigs or scaffolds using multiple assemblers (e.g. SOAPdenovo2 (41), SPAdes (42), Velvet (43) and Platanus (44)). Then, the best assembly result is selected according to N50, N75, the contig number, the length of the largest contig and the number of total bases and ambiguous bases. Thereafter, the reads are mapped to the best assembly result to check for mis-assemblies and evaluate the reads coverage.
- The final assembly result (i.e. best assembly) is used to estimate the completeness and contamination of the genome using checkM (45) and to perform further genomic component analysis.
- **Genomic component analysis:** Genomic component analysis involves CRISPR array recognition using PILER-CR (46), repetitive structure detection using TRF (47), non-coding RNA prediction using tRNAscanSE (48) and RNAmmer and gene prediction using Prodigal (49). The analysis is performed based on the final assembly result.
- **Gene annotation**: Predicted genes are annotated using several databases, including KEGG, GO (50), COG, NR (51), Swiss-Prot (52), AntiSMASH (53), MetaCyc (54), PHI (55), Pfam (56), CARD and VFDB (57).

### New species identification pipeline

For the new species identification pipeline (shown in Figure 4B), the new type strain genome sequence is used as the query in a similarity search against the gcType 16S rRNA gene and genome sequence reference database following the recommendations for the use of genome data in IJSEM.

First, 16S rRNA gene sequence(s) are extracted from the submitted query genome sequence. If a full-length 16S rRNA sequence of the same type strain sequenced using the Sanger method is available, it is compared with the 16S rRNA sequence extracted from the whole-genome assembly to ensure authenticity of the data.

Second, the 16S rRNA gene sequence is aligned to the gcType 16S rRNA gene database using the BLAST tool. Sequences with the highest similarity are used to estimate 16S rRNA gene completeness (58). Users are allowed to select a set of 16S rRNA gene sequences for further phylogenetic analyses from neighbouring sequences or sequences with a remote distance to serve as the reference.

Third, the submitted genome sequence is aligned to the gcType whole-genome reference database using Mash (59) to calculate the genome distance. Various genome similarity metrics, including ANIb (60), FastANI (61), orthoANIb and orthoANIu (62), are provided for the calculation of similarity between a submitted genome sequence and the selected sequences.

Finally, selected 16S rRNA gene sequences are aligned using MAFFT (63) or MUSCLE (64). Then, MEGA (65), FastTree (66) and RAxML (67) are used to perform phylogenetic analyses. For the selected genome sequences, 56 marker genes (68) are extracted and used to perform phylogenetic analyses.

**Figure 5.**
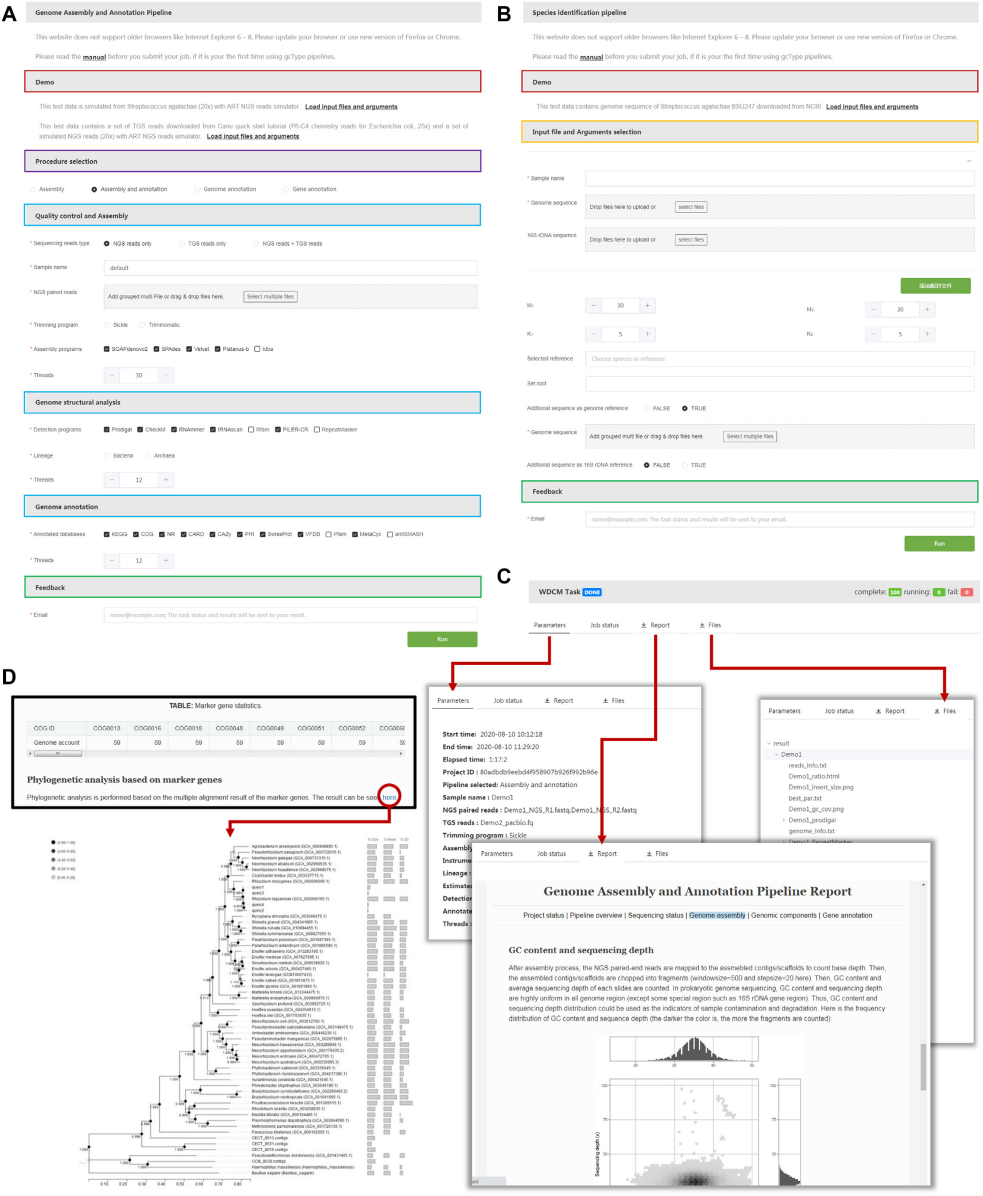
Submission pages and results of the analysis for the two pipelines, provided by gcType. (**A**) Submission page of a genome assembly and annotation pipeline. Different analytical tools and annotation databases are provided. (**B**) Submission page of a species identification pipeline. (**C**) Pages of the online results include parameters selected by the user, logs, report and output file. (**D**) An example of a phylogenetic analysis based on marker genes from a species identification pipeline.

Online services and results of analyses of these two pipelines are displayed in Figure 5A-5D.

## RESULTS AND DISCUSSION

The ability to accurately identify and organise microorganisms into appropriate taxonomic groups is essential for the functional research of microorganisms. With considerable advancements in genome sequencing and data analytics, genomics is the most powerful and efficient method for studying the origins, evolution and interactions of a species and the diversity of the microbial world. As the taxonomic representatives of various species, type strains are very essential resources for genome sequencing. Currently, type strains are preserved in internationally renowned culture collections such as Leibniz-Institut Deutsche Sammlung von Mikroorganismen und Zellkulturen GmbH (DSMZ), Japan Collection of Microorganisms (JCM)/RIKEN BioResource Center and American Type Culture Collection. The number of type strains and the diversity of species are increasing in culture collections across the world. The top five culture collections with the largest type strain deposition account for 88.02% of all validly published species, and 97.13% of all published species are represented in ten culture collections.

The operations of microbial collections have changed enormously over the past 20 years owing to the availability of multidisciplinary data as well as advances in analytical methods and bioinformatics. Traditional culture collections have made great efforts to explore the diversity of microorganisms and collect information on their genes, properties and products. For instance, with the joint effort of the Department of Energy (DOE) Joint Genome Institute (JGI) (69), DSMZ has already published 3,963 type strain genome sequences (Table 1). Large lists of published strain sequences are limited to culture collections that allocate large budgets to sequence, analyse and publish these sequences. Because this is not feasible for most collections, the GCM 10K project can assist them in getting their items sequenced and published while still being available for their repositories.

**Table 1.** Top 10 culture collections with the largest number of type strains

No.	Culture collection	Country	Preserved type strains^a^	Sequenced type strains
**1**	DSMZ	Germany	9106	3963
**2**	JCM	Japan	7200	824
**3**	ATCC	United States	4477	911
**4**	BCCM/LMG	Belgium	3513	385
**5**	NBRC	Japan	3369	716
**6**	KCTC	Korea	3243	220
**7**	CCUG	Sweden	3030	176
**8**	CIP	France	2784	61
**9**	NRRL	United States	1592	606
**10**	CGMCC	China	1558	145

^a^The number of type strains preserved in each culture collection was manually extracted from IJSEM. Type strains which were obtained from other culture collections by exchange of strains were not included.

The number of sequenced bacterial and archaeal genomes has grown exponentially in recent years. As a sequencing center, DOE JGI is currently the largest generator of type strain sequences and has published 3066 sequences to date, followed by WDCM, which has published 1049 genome sequences of type strains after two year of working with the GCM 10K sequencing project. The data collected in the GCM 10K project is the result of a collaboration of 22 global culture collections. The National Institute of Technology and Evaluation and the University of Tokyo rank third and fourth, generating 386 and 272 type strain genome sequences, respectively, with most of these strains coming from the Biological Resource Center/National Institute of Technology and Evaluation (NBRC) and JCM. The top ten sequencing centres contribute 42% of the total number of sequences, while the remaining 58% is contributed by 477 sequencing centres (Table 2).

**Table 2.** Sequencing efforts of global sequencing centres

No.	Sequencing centres	Number of publicly available type strain genomes
1	DOE Joint Genome Institute (JGI)	3066
2	WDCM GCM 10K project	1049
3	National Institute of Technology and Evaluation	386
4	University of Tokyo	272
5	Shanghai Majorbio Bio-pharm Technology Co.	183
6	J. Craig Venter Institute	147
7	Broad Institute	131
8	Washington University in St. Louis	125
9	Wellcome Trust Sanger Institute	97
10	Baylor College of Medicine	95
	Others	7784

**Figure 6.**
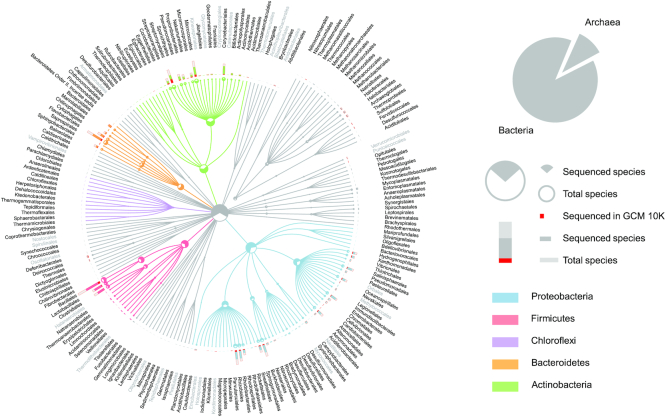
Current sequencing status of prokaryotic organisms by different orders. Top five phyla with the largest number of orders are highlighted. Published type strain genomes of the GCM 10K project are widely distributed in various orders. For orders that have more than nine species (nine is the median number of species of all orders), the order names are coloured in grey if <50% of species have type strain genome sequences, indicating more sequencing efforts should be focussed on these orders.

To date, whole-genome sequences of >13 962 type strains are publicly available. However, over one-fourth of all bacterial type strains have yet to be sequenced (Figure 6). Among the sequenced type strains, less than 21% (2,911) have high-quality, completed genomes. The remaining 11 051 genomes have been published as draft genomes, meaning that each one is composed of numerous contigs or scaffolds rather than a single contiguous and complete sequence. These draft sequences are valuable for some applications but do not permit the scientific community to fully study topics which require greater detail, such as structure, evolution and comparative genomics. Additional efforts to sequence, catalogue and characterise high-quality type strain genomes to provide a comprehensive coverage of all species with validly published names are urgently needed.

## FUTURE DIRECTIONS

Although taxonomic and phylogenetic analyses based on 16S rRNA gene sequences are commonly used methods for bacterial identification, the prevalence of genome-based taxonomy has become more prevalent as a result of the increasing availability of high-quality reference databases and user-friendly analysis pipelines. Taxonomists are required to provide ANI or dDDH comparisons to type strains when identifying isolates, proposing new species, or reclassifying accepted species. However, because many type strain genomes of species with validly published names remain un-sequenced, taxonomists require these additional type strain genomes so that their target strains can be analysed with appropriate scientific rigour. To address this critical need, the GCM 10K project is providing free sequencing services to complete these efforts, which are now complemented by the gcType platform.

In the future, gcType will continuously integrate data from various resources and publish updated results of the GCM 10K type strain sequencing project, making it a unique resource for comprehensive type strain genome information. gcType will also integrate data pertaining to genomic and phenotypic characteristics of a taxonomic group to help define associations between genomic and phenotypic characteristics and further predict metabolic features based on the combination of the integrated information. Finally, as the high-quality reference genomic data will provide accurate taxonomic and functional predictions for metagenomic data, genome assemblies from metagenomic samples will be integrated to expand the utility of the database for uncultured prokaryotic organisms.

## DATA AVAILABILITY

There are no access restrictions for the academic use of the platform. All public available genomic data or metadata are freely accessible. Both the 16S rRNA gene sequences and whole-genome sequences generated by the GCM 10K project have been continuously submitted to INSDC after data curation. Access to gcType is free at: http://gctype.wdcm.org/.
